# Effects of *Enteromorpha* polysaccharide-selenium on oxidative stress, immune stress, and intestinal health in broilers

**DOI:** 10.3389/fnut.2026.1796127

**Published:** 2026-04-28

**Authors:** Hongfei Li, Hao Lv, Zhiyong Rao, Wei Zhang, Yu Tang, Zhixiang Wang, Yongpeng Guo

**Affiliations:** 1 College of Animal Science and Technology, Henan Agricultural University, Zhengzhou, China; 2 College of Animal Science and Technology, China Agricultural University, Beijing, China

**Keywords:** antioxidant capacity, Enteromorpha polysaccharide-selenium, gut microbiota, inflammatory response, lipopolysaccharide

## Abstract

**Purpose:**

Intensive and large-scale poultry farming often induces oxidative and immune stress in broilers, compromising their health and production performance. In this study, a lipopolysaccharide (LPS) challenge model was established to induce oxidative and immune stress, and Enteromorpha polysaccharide-selenium (EP-Se) was evaluated as an organic selenium alternative to sodium selenite in broiler diets.

**Methods:**

A total of 192 one-day-old male Arbor Acres broilers were randomly assigned to four dietary treatments (*n* = 6 replicates per treatment, 8 birds per replicate): (1) SS, basal diet supplemented with 0.3 mg/kg selenium from sodium selenite; (2) LPS, SS diet plus LPS challenge; (3) EP-Se, basal diet supplemented with 0.3 mg/kg selenium from EP-Se; and (4) LSE, EP-Se diet plus LPS challenge. The experimental period lasted 21 days.

**Results:**

EP-Se supplementation significantly increased body weight, average daily gain (ADG), and average daily feed intake (ADFI) (*P* < 0.05), mitigating LPS-induced growth suppression. It enhanced antioxidant capacity by reducing malondialdehyde (MDA) levels, upregulating antioxidant gene expression, and increasing antioxidant enzyme activity (*P* < 0.05). EP-Se also alleviated LPS-induced inflammation by inhibiting activation of the TLR/NF-κB signaling pathway and reducing intestinal proinflammatory cytokine levels (*P* < 0.05). Furthermore, EP-Se improved intestinal health by modulating gut microbiota composition and increasing short-chain fatty acid concentrations (*P* < 0.05).

**Conclusion:**

Collectively, EP-Se exhibits potent antioxidant and anti-inflammatory effects and promotes gut health in LPS-challenged broilers. These findings provide a theoretical basis for developing EP-Se as a functional organic selenium additive for poultry feed.

## 1 Introduction

Modern broiler chicken farming models include industrialized, three-dimensional, ecological, family farm, and cooperative systems. Among these, industrialized farming is the most common, characterized by high automation, high production efficiency, and stable product quality (1, 2). However, broiler flocks raised under this model are prone to oxidative and immune stress (3, 4). Environmental factors such as high temperature, poor ventilation, and feed mycotoxin contamination can elevate reactive oxygen species (ROS) production beyond the birds’ antioxidant capacity, resulting in oxidative stress (3). Meanwhile, physical stressors arising from transportation, flock transfer, and vaccination, together with the risk of pathogen transmission in high-density conditions, can compromise immune function and induce immune stress (5, 6). Hence, effective prevention or mitigation of oxidative and immune stress has become a key focus in broiler production research.

Selenium, an essential trace element, is widely used in broiler feed because it enhances antioxidant defenses, supports immune function, promotes growth and development, improves meat quality, and increases feed utilization efficiency (7, 8). Traditional inorganic selenium additives, such as sodium selenite, are cost-effective but limited by low bioavailability (9, 10). To address these shortcomings, organic selenium sources such as selenium yeast, selenomethionine, and selenium polysaccharides have attracted increasing attention for their higher bioavailability, lower toxicity, and superior absorption. These compounds significantly improve broiler growth performance, antioxidant capacity, and immune response (11, 12).

Polysaccharide-selenium (selenopolysaccharide) is a novel organic selenium compound with notable advantages and research potential (13). The conjugation of selenium with polysaccharides enhances both selenium bioavailability and polysaccharide biological activity (14). Compared with selenium or polysaccharides used alone, selenopolysaccharides exert stronger antioxidant effects and more effectively improve immune responses and disease resistance, demonstrating a synergistic interaction between the two components (15). Moreover, compared with inorganic selenium additives, polysaccharide-selenium exhibits lower toxicity, and its solvent-free production process reduces environmental pollution (16, 17).

Enteromorpha, a widely distributed green alga, is rich in polysaccharides, proteins, and minerals and offers considerable development potential. Previous studies have shown that Enteromorpha polysaccharides possess strong antioxidant, immunoregulatory, and antitumor properties (18, 19). However, research on its conjugation with selenium to form selenopolysaccharides in broiler nutrition and production remains limited.

Based on the aforementioned background, the present study was designed to test the following hypotheses: (i) dietary supplementation with Enteromorpha polysaccharide-selenium (EP-Se) would exert superior protective effects against oxidative and immune stress compared with sodium selenite in broiler chickens; (ii) EP-Se would alleviate lipopolysaccharide (LPS)-induced hepatic and intestinal injury through modulation of redox-sensitive signaling pathways and suppression of inflammatory responses; and (iii) the beneficial effects of EP-Se on intestinal health would be associated with alterations in gut microbiota composition and enhanced production of short-chain fatty acids (SCFAs). To verify these hypotheses, an oxidative and immune stress model was established using LPS challenge, followed by evaluation of growth performance, antioxidant capacity, inflammatory parameters, intestinal morphology, and cecal microbiota in broilers fed diets supplemented with EP-Se or sodium selenite. These findings are expected to provide a theoretical basis for the application of EP-Se as a novel organic selenium feed additive in broiler production.

## 2 Materials and methods

### 2.1 Experimental animals and treatment

This study was approved and supervised by the Animal Ethics Committee of Henan Agricultural University (Approval No. HENAU-2023-015). EP-Se used in this study was mainly composed of rhamnose, glucose, xylose, galactose, and mannose at a molar ratio of 1:1.25:0.06:0.02:0.01. The polysaccharide content was ≥ 35% and the selenium content was ≥ 1.8%. A total of 192 one-day-old male Arbor Acres broilers were randomly assigned to four dietary treatments (n = 6 replicates per treatment, 8 broilers per replicate): (1) SS, basal diet supplemented with 0.3 mg/kg selenium from sodium selenite; (2) LPS, SS diet plus LPS challenge; (3) EP-Se, basal diet supplemented with 0.3 mg/kg selenium from EP-Se; and (4) LSE, EP-Se diet plus LPS challenge. Broilers were maintained under controlled environmental conditions (45-60% relative humidity, 17L:7D photoperiod) with *ad libitum* access to feed and water. Ambient temperature was maintained at 35°C during the first week and gradually decreased by 2°C per week until reaching 27C°. The basal diet without selenium supplementation was mixed with sodium selenite or EP-Se at a supplemental level of 0.3 mg Se/kg diet. The selenium content in the experimental diets was measured by hydride generation atomic fluorescence spectrometry and confirmed to be 0.33 mg/kg for the SS diet and 0.32 mg/kg for the EP-Se diet. The control diet was formulated to meet NRC (1994) nutrient requirements (Table 1). Vaccination was performed according to standard protocols: Newcastle disease–infectious bronchitis combined vaccine at d7, infectious bursal disease vaccine at d14, and Newcastle disease booster at d21. The LPS-challenged groups (LPS and LSE) received intramuscular injections of 1 mg/kg body weight (BW) LPS (E. coli serotype 055: B55; Sigma, Saint Louis, MO, United States) on days 16, 18, and 20, whereas the SS and EP-Se groups were injected with sterile saline. The experimental period lasted 21 days.

**TABLE 1:** The compositions of the diet and the nutritional level (air-dried basis,%).

Item	Content, %
Corn	54.54
Soybean meal	33.80
Corn protein flour	5.00
Dicalcium phosphate	1.00
Limestone	1.50
NaCl	0.30
Premix^1^	0.60
Soybean oil	2.80
L-Lysine sulfate (70%)	0.16
DL-methionine (99%)	0.15
Calcium bicarbonate	0.15
Total	100.00
Nutrient composition	
Avian metabolic energy, MJ/kg	12.52
Crude protein, %	23.04
Calcium, %	0.91
Total phosphorus, %	0.70
Lysine, %	1.20
Methionine, %	0.50

^1^The following was supplied per kg complete diet: vitamin A, 15,000 IU; vitamin D3, 4,500 IU; vitamin E, 40 IU; vitamin K3, 3 mg; vitamin B_1_, 1.8 mg; vitamin B_2_, 7.5 mg; vitamin B_6_, 4.8 mg; vitamin B_12_, 0.03 mg; biotin, 0.24 mg; folic acid, 1.2 mg; pantothenic acid, 12 mg; niacin, 45 mg; choline chloride, 800 mg; Phytase 100 mg; Cu, 16 mg; Zn, 110 mg; Fe, 20 mg; Mn, 120 mg; I, 1.25 mg.

### 2.2 Growth performance and sample collection

On day 21, all broilers were fasted for 8 h. Body weight and feed intake were recorded to calculate average daily feed intake (ADFI), average daily gain (ADG), and feed conversion ratio (FCR). One broiler with an average BW was selected from each replicate for sample collection. Blood was drawn from the inferior pterygoid vein, centrifuged, and the serum stored at -20°C. Before slaughter, broilers were anesthetized with pentobarbital sodium. After slaughter, the liver, spleen, cecal contents, and 4 cm segments of the duodenum, jejunum, and ileum were collected. Each intestinal segment was divided into two parts-one fixed in paraformaldehyde for histology and the other frozen in liquid nitrogen. All samples were stored at -80°C for subsequent analyses.

### 2.3 Assay of antioxidant and immune indices

Blood samples were thawed at room temperature. Tissue samples were homogenized in ice-cold saline and centrifuged, and supernatants were stored at -20°C. Serum and tissue supernatants were analyzed for total antioxidant capacity (T-AOC), catalase (CAT), thioredoxin reductase (TrxR), glutathione peroxidase (GSH-Px), MDA, tumor necrosis factor-α (TNF-α), interleukin-1β (IL-1β), and interleukin-6 (IL-6) using commercial ELISA kits (Shanghai Meilian Biology Technology, Shanghai, China).

### 2.4 Histological evaluation

Sections of the duodenum, jejunum, and ileum were fixed in 4% paraformaldehyde, embedded in paraffin, and stained with hematoxylin and eosin (H&E). Villus height (VH) and crypt depth (CD) were measured in 5–6 villi per slide using a light microscope and Image-Pro Plus software (Image Pro Plus; Media Cybernetics, Bethesda, MD, United States), and the VH/CD ratio was calculated. The analytical procedure followed the method described by Liao et al. (20).

### 2.5 mRNA expression analysis

Total RNA was extracted from liver and duodenum tissues using Trizol reagent. RNA concentration and purity were assessed using a NanoDrop 2000 spectrophotometer (Thermo Fisher Scientific Co., Waltham, MA). Complementary DNA (cDNA) was synthesized with the HiScript reverse transcription kit (Vazyme Biotech, Nanjing, China). Quantitative real-time PCR (RT-qPCR) was performed using SYBR Green Master Mix (Vazyme Biotech, Nanjing, China) on a Bio-Rad detection system (BioRad Laboratories, Mississauga, ON, Canada), with GAPDH as the internal reference gene. The procedure followed the method described by Liu et al. (21). Primer sequences for target and reference genes are shown in Table 2.

**TABLE 2:** Gene special primers used in the real-time quantitative reverse transcription PCR.

Gene name	Accession number	Primer sequence (5′–3′)
GPX1	NM_001277853.1	Forward 5′-ACGGCGCATCTTCCAAAG-3′ Reverse 5′-TGTTCCCCCAACCATTTCTC-3′
GPX2	NM_001277854.1	Forward 5′- ATCGCCAAGTCCTTCTACGA-3′ Reverse 5′-ACGTTCTCGATGAGGACCAC-3′
GPX3	NM_001163232.1	Forward 5′- CCTGCAGTACCTCGAACTGA-3′ Reverse 5′-CTTCAGTGCAGGGAG GATCT-3′
GPX4	AF498316	Forward 5′- CTTCGTCTGCATCATCACCAA-3′ Reverse 5′-TCGACGAGCTGAGTGTAA TTCC-3′
Txnrd1	NM_001030762.2	Forward 5′- TACGCCTCTGGGAAATTCGT-3′ Reverse 5′-CTTGCAAGGCTTGTCCCAGTA-3′
Txnrd2	NM_001122691.1	Forward 5′-GCTCTTAAAGATGCCCAGCACTAC-3′ Reverse 5′-GAACAGCTTGAGCCATCACAGA-3′
Txnrd3	NM_001122777.1	Forward 5′- CCTGGCAAAACGCTAGTTGT G-3′ Reverse5′-CGCACCATTACTGTGACATCTAGAC-3′
SEL-T	NM_001006557.3	Forward 5′- AGGAGTACATGCGGGTCATCA-3′ Reverse5′-GACAGACAGGAAGGATGCTATGTG-3′
SEL-H	BX932816.2	Forward 5′- CATCGAGCACTGCCGTAG -3′ Reverse 5′- GACACCTCGAAGCTGTTCCT -3′
SEL-O	NM_001115017.1	Forward 5′- CCAGCGTTAACCGGAATGAT -3′ Reverse 5′- ATGCGCCTCCTGGATTTCT -3′
SEL-W1	NM_001166327.1	Forward 5′- TGGTGTGGGTCTGCTTTACG -3′ Reverse 5′- CCAAAGCTGGAAGGTGCAA -3′
SEL-M	CR390234.1	Forward 5′- AAGAAGGACCACCCAGACCT -3′ Reverse 5′- GCTGTCCTGTCTCCCTCATC -3′
HQO-1	NM_001277621.1	Forward 5′- GGCAATGGCAGCAGCAG -3′ Reverse 5′- AAGCACTCGGGGTTCTTGAG -3′
HO-1	NM_205344.1	Forward 5′- TCCACGAGTTCAAGCTGGTC -3′ Reverse 5′- AGCCTCAGGACATGGGATCT -3′
SOD	NM_205064.1	Forward 5′- AAATGGGTGTACCAGCGCA -3′ Reverse 5′- CTTTGCAGTCACATTGCCGA -3′
Nrf2	NM_205117.1	Forward 5′- CTTCAGGGGTAGCAAGGTATGA -3′ Reverse 5′- TTCCCAGTTCGGTGCAGAAG -3′
GCLM	NM_001007953.1	Forward 5′- CCAGAACGTCAAAGCACACG -3′ Reverse5′- TCCTCCCATCCCCCAGAAAT -3′
MyD88	NM_001030962	Forward 5′- CAGAAAGACCTTCAGTTTGT-3′ Reverse 5′-AATGACGACCACCATCCTCC -3′
CHUK	NM_001012904.2	Forward 5′- AACTCCTGGAGATGGGGAAAAC-3′ Reverse 5′- TCTCTTGCCTCCTGCAACAT -3′
TARF6	XM_040673311.2	Forward 5′- CCGAAACTGAAAGGCGTTCT-3′ Reverse 5′- TATCCAAGTCTCGGCCTCCA -3′
IL-6	NM_204628.1	Forward 5′-CGTTTATGGAGAAGACCGTGAG -3′ Reverse 5′- CGTTTATGGAGAAGACCGTGAG -3′
IL-8	NM_205498.1	Forward5′- ATGAACGGCAAGCTTGGAGCTG -3′ Reverse 5′-TCCAAGCACACCTCTCTTCCATCC-3′
NF-κB	NM_205129.1	Forward5′-GTGTGAAGAAACGGGAACTG-3′ Reverse 5′-GGCACGGTTGTCATAGATGG-3′
TLR-4	NM_01030693.1	Forward5′-AGTCTGAAATTGCTGAGCTCAAAT-3′ Reverse 5′-GCGACGTTAAGCCATGGAAG-3′
iNOS	NM_204961.1	Forward 5′- CCTGTACTGAAGGTGGCTATTGG-3′ Reverse 5′-AGGCCTGTGAGAGTGTGCAA-3′
GADPH	NM_204305.1	Forward 5′- TGCTGCCCAGAACATCATCC-3′ Reverse 5′-ACGGCAGGTCAGGTCAACAA-3′

### 2.6 16 S rRNA sequencing of cecal microbiota

Following the manufacturer’s directions, total bacterial genomic DNA samples were extracted using the Fast DNA SPIN extraction kits (MP Biomedicals, Santa Ana, CA, United States). The quantity and quality of extracted DNA were measured using a NanoDrop ND-1,000 spectrophotometer (Thermo Fisher Scientific, Waltham, MA, United States) and agarose gel electrophoresis, respectively. Illumina MiSeq sequencing and general data analyses were performed by a commercial company (Majorbio, Shanghai, China). The 16S V4 region of 16S rRNA genes were amplified by the specific primer with the barcode (338F: 5′-ACTCCTACGGGAGGCAGCAG-3′; 806R: 5′-GGACTACHVGGGTWTCTAAT-3′) by thermocycler PCR system (GeneAmp 9700, ABI, United States). According to the manufacturer’s instructions, the PCR products were extracted from a 2% agarose gel, further purified using the AxyPrep DNA Gel Extraction Kit (Axygen Biosciences, Union City, CA, United States) and quantified using QuantiFluor™-ST (Promega, United States). Purified amplicons were pooled in equimolar and paired-end sequenced (2 × 300) on an Illumina MiSeq platform (Illumina, San Diego, United States) according to the standard protocols by Majorbio Bio-Pharm Technology Co., Ltd. (Shanghai, China).

### 2.7 Determination of short-chain fatty acids (SCFAs)

In accordance with the method described by Liu et al. (22), 1 g of cecal contents was diluted with 15 mL of double-distilled water and centrifuged at 5,000 × g for 10 min. Subsequently, 1 mL of the supernatant was transferred into a centrifuge tube and mixed with 0.2 mL of pre-cooled 25% (w/v) metaphosphoric acid for protein precipitation. The mixture was incubated at 4°C overnight, followed by centrifugation at 12,000 × g for 10 min. The resulting supernatant was collected and filtered through a 0.22 μm membrane prior to analysis. Short-chain fatty acids, including acetate, propionate, butyrate, and valerate, were quantified using a Thermo Scientific DIONEX ICS-5000 + ion chromatography system equipped with a conductivity detector and a Dionex IonPac™ AH11-HC analytical column. Samples were introduced via an autosampler (25 μL injection volume), and separation was achieved by gradient elution with 1.5 mM/100 mM sodium hydroxide as the mobile phase.

### 2.8 Statistical analysis

Growth performance was analyzed using replicates as experimental units (*n* = 6). Other parameters were assessed from six randomly selected samples per replicate (*n* = 6). A 2 × 2 factorial design was applied. The main effects of selenium source and LPS challenge, as well as their interaction, were analyzed using two-way ANOVA (IBM SPSS 21.0). When the interaction effect was significant, Bonferroni’s test was used for *post hoc* multiple comparisons between means. Figures were produced using GraphPad Prism 8.0. Raw 16S rRNA gene sequencing data were quality-filtered using fastp and merged with FLASH. Operational taxonomic units (OTUs) were clustered at 97% sequence similarity using UPARSE, with chimeric sequences removed during the clustering process. Taxonomic annotation was performed against the SILVA 138 database with a confidence threshold of 0.7. Alpha diversity indices, including Chao1, ACE, Shannon, and Simpson, were analyzed using two-way analysis of variance (ANOVA). Beta diversity was assessed based on Bray–Curtis distances, followed by principal coordinates analysis (PCoA) and permutational multivariate analysis of variance (PERMANOVA) with 999 permutations. Differential taxa were identified using Kruskal–Wallis H test, and pairwise comparisons were conducted using Mann-Whitney U Test. Benjamini-Hochberg method was used for FDR correction, and *P* < 0.05 was considered statistically significant. Data are presented as mean ± standard error of the mean (SEM), with statistical significance set at *P* < 0.05.

## 3 Results

### 3.1 Effects of EP-Se on growth performance in LPS-challenged broilers

**FIGURE 1:**
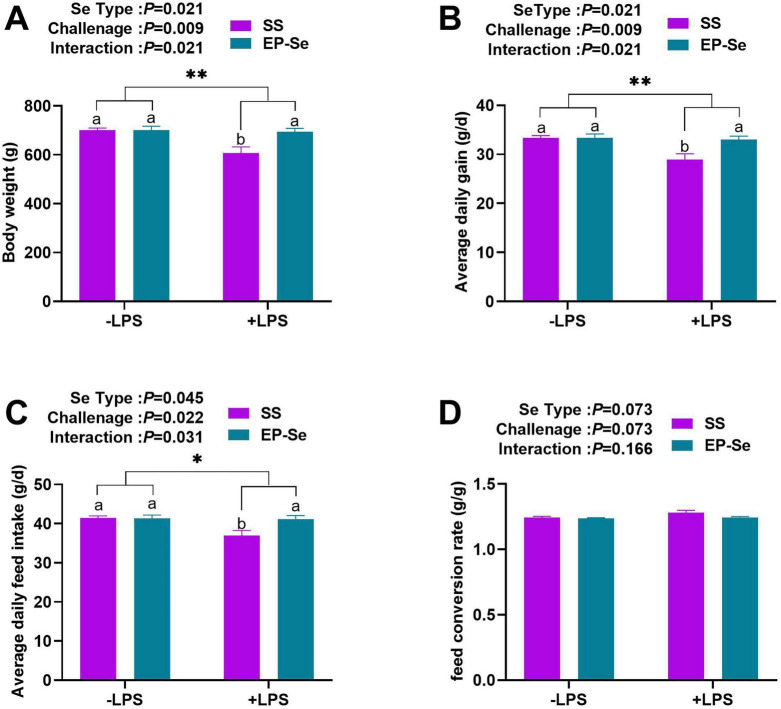
Dietary replacement of SS with EP-Se on the growth performance of LPS-challenged broilers. Data analysis of broiler body weight **(A)**, average daily gain **(B)**, average daily feed intake **(C)**, and feed-to-gain ratio **(D)**. Values are means (*n* = 6) with their standard errors represented by vertical bars. *P < 0.05, **P < 0.01 showed that the main effect of LPS was significant. ^a,b^Different letters above bars indicate significant difference (*P* < 0.05).

Figure 1 shows a significant interaction between dietary selenium source and LPS challenge on BW, ADG, and ADFI (*P* < 0.05). Multiple comparisons indicated that the + LPS/SS group had significantly lower BW, ADG, and ADFI than the −LPS/SS group (*P* < 0.05), whereas no significant differences were observed between the + LPS/EP-Se and −LPS/EP-Se groups (*P* > 0.05). Moreover, BW, ADG, and ADFI were significantly higher in the + LPS/EP-Se group than in the + LPS/SS group (*P* < 0.05). These findings suggest that LPS challenge impaired growth performance, whereas EP-Se supplementation mitigated this effect. These findings indicate that LPS-induced growth impairment occurred only under sodium selenite (SS) supplementation, whereas EP-Se supplementation effectively mitigated the adverse effects of LPS challenge on growth performance.

### 3.2 Effects of EP-Se on hepatic mRNA expression of antioxidant factors

Table 3 shows that LPS significantly downregulated the mRNA expressions of *GPx2*, *GPx4*, *SEL-H*, *HO-1* and *NQO1* (*P* < 0.05). Compared with SS, EP-Se significantly up-regulated *GPx2*, *GPx3*, *NQO1*, *Nrf2* and *SOD* expression (*P* < 0.05). A significant interaction between selenium source and LPS challenge was detected for *SEL-T* expression (*P* < 0.05). Multiple comparisons revealed that *SEL-T* mRNA levels were significantly lower in the + LPS/SS group than in the −LPS/SS group (*P* < 0.05), whereas no difference was observed between the −LPS/EP-Se and + LPS/EP-Se groups (*P* > 0.05). In addition, *SEL-T* mRNA expression was significantly higher in the + LPS/EP-Se group than in the + LPS/SS group (*P* < 0.05).

**TABLE 3:** Dietary replacement of SS with EP-Se on mRNA expression of antioxidant factors in liver of LPS-challenged broilers (*n* = 6).

Item^1^	SS^2^		EP-Se^3^		SEM	*P*-value		
	LPS (-)	LPS (+)	LPS (-)	LPS (+)		SS/EP-Se	± LPS	Interaction
GPX1	1.000	0.835	1.450	1.238	0.212	0.058	0.385	0.914
GPX2	1.000	0.766	2.787	1.012	0.450	0.035	0.037	0.102
GPX3	1.000	0.709	2.044	2.076	0.253	< 0.001	0.614	0.531
GPX4	1.000	0.581	1.035	0.662	0.157	0.717	0.020	0.885
TXNRD1	1.000	0.940	1.212	1.148	0.184	0.266	0.739	0.989
TXNRD2	1.000	0.717	1.205	0.949	0.140	0.135	0.069	0.923
TXNRD3	1.000	0.974	1.213	1.166	0.102	0.061	0.732	0.921
SEL-T	1.000^a^	0.537^b^	1.087^a^	1.079^a^	0.156	0.007	0.037	0.043
SEL-H	1.000	0.602	1.133	0.697	0.135	0.407	0.006	0.891
HO-1	1.000	0.572	1.040	0.728	0.129	0.455	0.009	0.657
NQO-1	1.000	0.844	1.363	1.028	0.060	< 0.001	0.001	0.153
Nrf2	1.000	0.603	2.284	2.176	0.321	< 0.001	0.440	0.658
SOD	1.000	0.759	1.058	1.058	0.065	0.013	0.081	0.080

^a,b^Means with no common superscript within each row are significantly different (*P* < 0.05).

^1^GPX1-4, glutathione peroxidases1-4; TXNRD1-3, thioredoxin reductases 1-3; SEL-T, selenoprotein T; SEL-H, selenoprotein H; HO-1, heme oxygenase-1; NQO1, NAD(P)H: quinone oxidoreductase 1; Nrf2, Nuclear factor erythroid 2-related factor 2; SOD, superoxide dismutase.

^2^SS, basal diet supplemented with Sodium Selenite.

^3^EP-Se, basal diet supplemented with Enteromorpha Polysaccharide Selenide.

### 3.3 Effect of EP-Se on antioxidant capacity in LPS-challenged broilers

**FIGURE 2:**
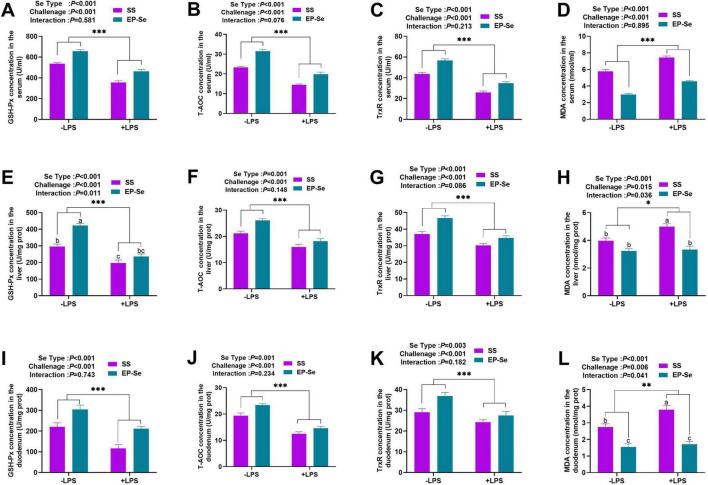
Dietary replacement of SS with EP-Se on the antioxidant parameters of different organs in LPS-challenged broilers. Twelve bar graphs labeled **(A–L)** compare the effects of two selenium types (SS in purple, EP-Se in teal) and LPS challenge (+LPS, −LPS) on various antioxidant and oxidative stress markers in serum **(A–D)**, liver **(E–H)**, and duodenum **(I–L)**. Values are means (*n* = 6) with their standard errors represented by vertical bars. *P < 0.05, **P < 0.01, ***P < 0.001 showed that the main effect of LPS was significant. ^a,b,c^Different letters above bars indicate significant difference (*P* < 0.05).

As illustrated in Figure 2, LPS challenge significantly decreased the activities of GSH-Px, T-AOC, and TrxR and increased MDA concentrations in the SS group (*P* < 0.05), whereas these effects were attenuated in the EP-Se group. EP-Se supplementation significantly enhanced antioxidant enzyme activities and reduced MDA levels regardless of LPS challenge (*P* < 0.05). Significant interactions between selenium source and LPS challenge were observed for hepatic GSH-Px activity as well as MDA concentrations in the liver and duodenum (*P* < 0.05). Multiple comparisons showed that hepatic GSH-Px activity in the + LPS/EP-Se group was significantly higher than that in the + LPS/SS group (*P* < 0.05) and did not differ from the −LPS/SS group (*P* > 0.05). Similarly, MDA levels in the liver and duodenum were significantly lower in the + LPS/EP-Se group than in the + LPS/SS group (*P* < 0.05) and were comparable to those in the −LPS/SS group (*P* > 0.05). These results indicate that the changes in hepatic GSH-Px activity and hepatic/duodenal MDA concentrations depended on the combined effect of selenium source and LPS challenge.

### 3.4 Effects of EP-Se on duodenal mRNA expression of inflammatory genes in LPS-challenged broilers

Table 4 indicates that LPS stimulation markedly increased *IL-6* and *iNOS* expression in the duodenum (*P* < 0.05), whereas EP-Se supplementation significantly reduced their expression levels (*P* < 0.05). A significant interaction between selenium source and LPS challenge was also observed for *IL-8* expression (*P* < 0.05). The + LPS/EP-Se group showed lower *IL-8* mRNA expression than the + LPS/SS group (*P* < 0.05), with no difference relative to the −LPS/SS group (*P* > 0.05). Meanwhile, LPS stimulation upregulated *TLR4*, *MYD88*, *CHUK*, *TRAF6*, and *NF-*κ*B* expression (P < 0.05), whereas EP-Se supplementation downregulated *TLR4*, *MYD88*, *TRAF6*, and *NF-*κ*B* expression (*P* < 0.05).

**TABLE 4:** Dietary replacement of SS with EP-Se on the relative mRNA expression of genes in Toll-like receptor pathways in duodenum of LPS-challenged broilers (*n* = 6).

Item^1^	SS^2^		EP-Se^3^		SEM	*P*-value		
	LPS (-)	LPS (+)	LPS (-)	LPS (+)		SS/EP-Se	± LPS	Interaction
TLR4	1.000	1.451	0.873	0.931	0.122	0.015	0.050	0.122
MYD88	1.000	1.389	0.881	1.154	0.055	0.005	< 0.01	0.306
CHUK	1.000	1.262	0.944	1.079	0.068	0.092	0.008	0.356
TRAF6	1.000	1.550	0.725	0.974	0.104	0.001	0.001	0.162
NF-κB	1.000	1.257	0.747	1.032	0.046	< 0.01	<0.01	0.762
iNOS	1.000	1.394	0.806	0.869	0.103	0.002	0.037	0.122
IL-6	1.000	2.163	0.882	1.009	0.263	0.026	0.024	0.063
IL-8	1.000b	2.896^a^	0.987^b^	1.003^b^	0.428	0.038	0.040	0.037

^a,b^ Means with no common superscript within each row are significantly different (*P* < 0.05).

^1^TLR4, toll-like receptor 4; MYD88, myeloid differentiation primary response 88; CHUK, inhibitor of nuclear factor kappa B kinase complex; TRAF6, TNF receptor associated factor 6; NF-κB, nuclear factor kappa B; iNOS, nitric oxide synthase 2; IL-6, interleukin-6; IL-8, interleukin-8.

^2^SS, basal diet supplemented with Sodium Selenite.

^3^EP-Se, basal diet supplemented with Enteromorpha Polysaccharide Selenide.

### 3.5 Effects of EP-Se on inflammatory cytokine levels in LPS-challenged broilers

**FIGURE 3:**
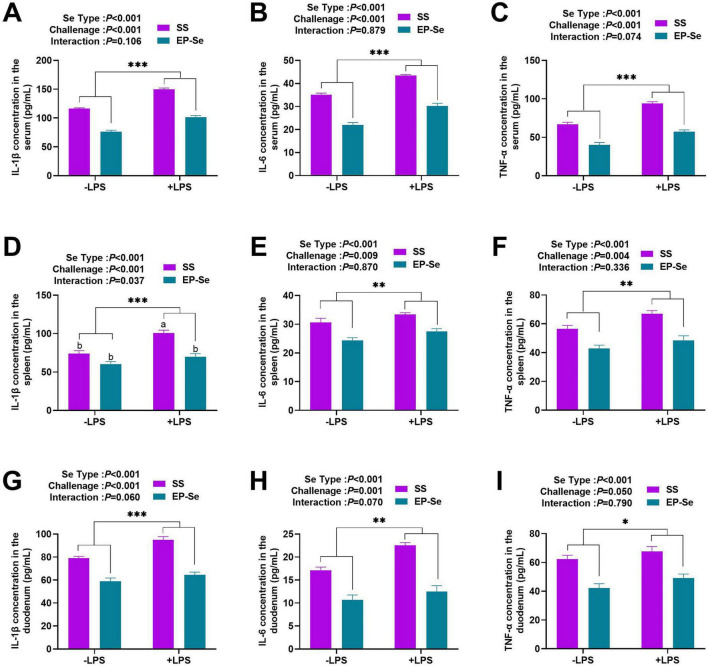
Dietary replacement of SS with EP-Se on the inflammatory factor levels in different organs of LPS-challenged broilers. Nine grouped bar charts labeled panels **A** to **I** compare IL-1β, IL-6, and TNF-α concentrations in serum **(A–C)**, spleen **(D–F)**, and duodenum **(G–I)** from SS and EP-Se groups under −LPS and +LPS conditions. Values are means (*n* = 6) with their standard errors represented by vertical bars. *P < 0.05, **P < 0.01, ***P < 0.001 showed that the main effect of LPS was significant. ^a,b^Different letters above bars indicate significant difference (*P* < 0.05).

As shown in Figure 3, LPS administration significantly increased IL-1β, IL-6, and TNF-α concentrations in the serum, spleen, and duodenum (*P* < 0.05). EP-Se treatment significantly reduced these cytokine levels (*P* < 0.05). An interaction between selenium source and LPS challenge affected spleen IL-1β levels (*P* < 0.05). Multiple comparisons indicated that spleen IL-1β concentrations were lower in the + LPS/EP-Se group than in the + LPS/SS group (*P* < 0.05), with no difference compared to the −LPS/SS group (*P* > 0.05).

### 3.6 Effects of EP-Se on intestinal morphology in LPS-challenged broilers

**FIGURE 4:**
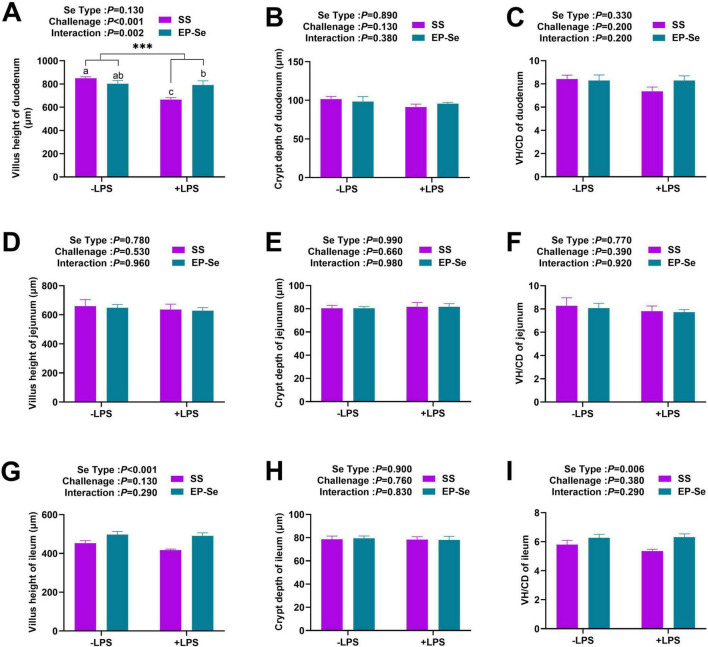
Dietary replacement of SS with EP-Se on the intestinal morphology of LPS-challenged broilers. Nine grouped bar graphs display villus height, crypt depth, and villus height to crypt depth ratio (VH:CD) of duodenum **(A–C)**, jejunum **(D–F)**, and ileum **(G–I)** from SS and EP-Se groups under −LPS and +LPS conditions. Values are means (*n* = 6) with their standard errors represented by vertical bars. ***P < 0.001 showed that the main effect of LPS was significant. ^a,b,c^Different letters above bars indicate significant difference (*P* < 0.05).

As presented in Figure 4, EP-Se supplementation significantly increased ileal VH and the VH-to-CD ratio (*P* < 0.05). A significant interaction between selenium source and LPS challenge was observed for duodenal VH (*P* < 0.05). Multiple comparisons revealed that the duodenal VH in the + LPS/EP-Se group was significantly higher than that in the + LPS/SS group (*P* < 0.05), with no significant difference observed compared with the −LPS/SS group (*P* > 0.05).

### 3.7 Effects of EP-Se on cecal microbiota composition in LPS-challenged broilers

**FIGURE 5:**
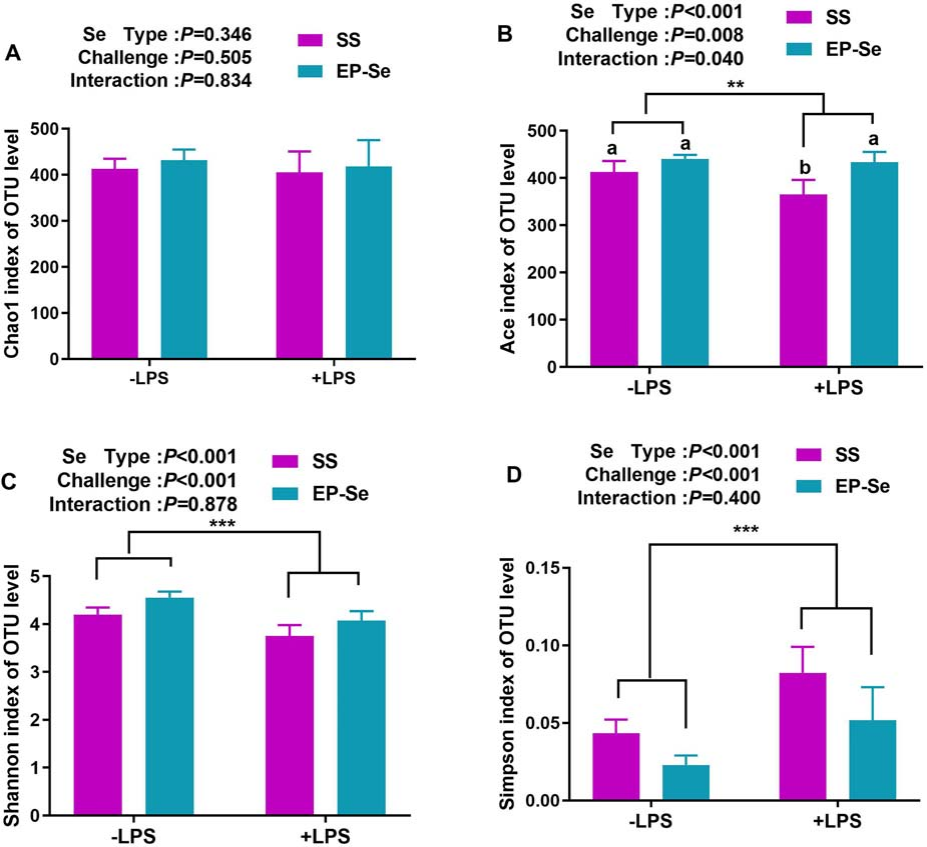
EP-Se improved cecal microbiota diversity in broilers challenged with LPS on OTU level. **(A)** Chao index; **(B)** Ace index; **(C)** Shannon index; **(D)** Simpson index. Values are means (*n* = 6) with their standard errors represented by vertical bars. **P < 0.01, ***P < 0.001 showed that the main effect of LPS was significant. ^a,b^Different letters above bars indicate significant difference (*P* < 0.05).

**FIGURE 6:**
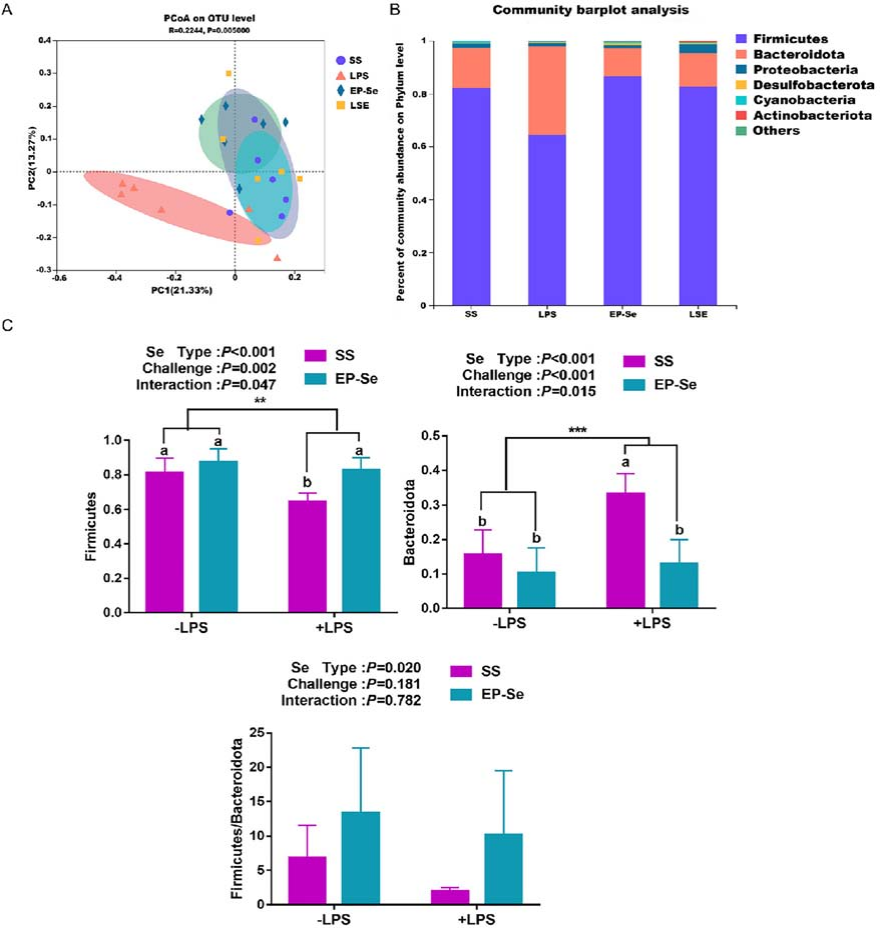
EP-Se improved cecal microbiota diversity in broilers challenged with LPS based on OTU level. **(A)** The principal co-ordinates analysis plot. **(B)** Microbial composition at the phylum level; **(C)** Composition differences of Firmicutes and Bacteroidota among groups. Values are means (*n* = 6) with their standard errors represented by vertical bars. **P < 0.01, ***P < 0.001 showed that the main effect of LPS was significant. ^a,b^Different letters above bars indicate significant difference (*P* < 0.05).

**FIGURE 7:**
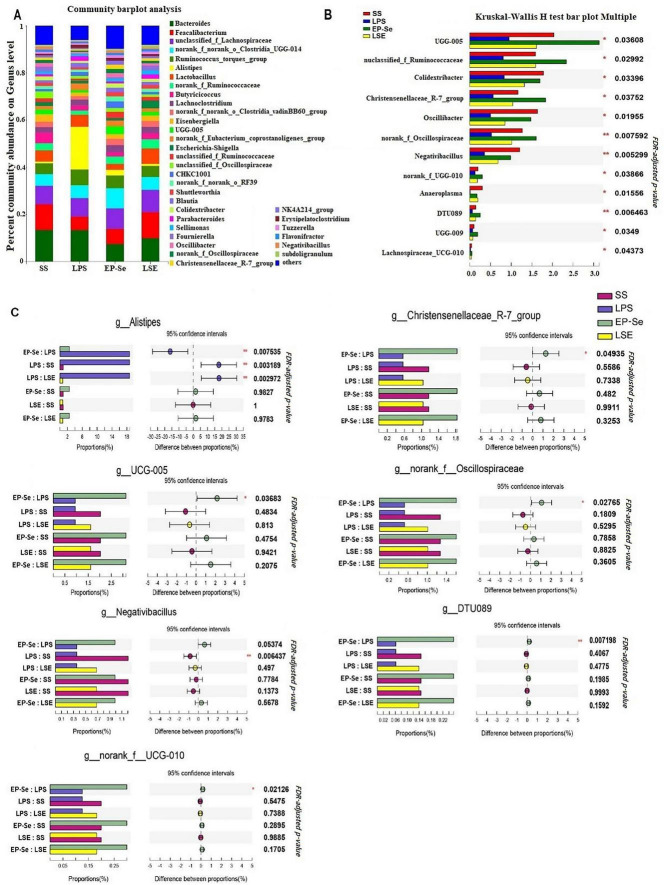
Effect of Se-EP on cecal microbiota of broilers stimulated by LPS at genus level. **(A)** Microbial composition at the genus level. **(B)** Compare the different microorganisms at the genus level of each group. **(C)** Changes in the intestinal microflora of chicken in different groups at the genus level. SS: SS/-LPS group; LPS: SS/ + LPS group; EP-Se: EP-Se/-LPS group; LSE: EP-Se/ + LPS group. *P < 0.05, **P < 0.01; *n* = 6.

EP-Se reshaped the cecal microbial community structure. There was no significant interaction between selenium source and LPS challenge on the Chao1 index (*P* > 0.05; Figure 5A). A significant interaction between selenium source and LPS challenge was observed for the Ace index. The +LPS/EP-Se group had a higher Ace index than the +LPS/SS group (*P* < 0.05), with no significant difference compared with the −LPS/SS group (*P* > 0.05; Figure 5B). Following LPS exposure, α-diversity was decreased in the SS group, as reflected by a reduced Shannon entropy and an increased Simpson dominance (*P* < 0.05). In contrast, EP-Se supplementation mitigated these LPS-induced changes (*P* < 0.05; Figures 5C,D). By Bray-Curtis principal component analysis, there were significant differences in microbiota in LPS group compared with the three other groups (Figure 6A). Community composition at the phylum level indicated that the dominant microbiota were Firmicutes (64.5–86.5%), Bacteroides (10.7–33.4%), Proteobacteria (1.1–3.7%), and Desulfobacterota (0.3–0.8%) (Figure 6B). Two-way ANOVA comparison of the different treatment groups showed that the addition of EP-Se significantly increased the ratio of Firmicutes to Bacteroides at the phylum level (*P* < 0.05). Selenium type and LPS stimulation had significant interaction on the levels of Firmicutes and Bacteroidetes (*P* < 0.05). By multiple comparisons, the relative abundance of Firmicutes was significantly decreased and that of Bacteroidetes was significantly increased in the + LPS/SS group compared to the -LPS/SS group. But these indexes were no significant difference between -LPS/EP-Se group and + LPS/EP-Se group (*P* > 0.05) (Figure 6C). Cecal microbial community composition analysis showed that at the genus level, the dominant genera in the SS, LPS, EP-Se, and LSE groups included *Bacteroides, Faecalibacterium, unclassified_f_Lachnospiraceae, norank_f_norank_o_Clostridia_UCG-014, Ruminococcus_torques_group, Alistipes, and Lactobacillus*. Community barplot analysis revealed distinct differences in microbial structure among the LPS group and the SS, EP-Se, and LSE groups, with notably elevated relative abundance of *Alistipes* in the LPS group (Figure 7A). Multiple comparisons of Kruskal-Wallis H test identified 12 genera with significant differences among the four groups (*P* < 0.05), including *UCG-005, unclassified_f_Ruminococcaceae, Colidextribacter, Christensenellaceae_R-7_group, Oscillibacter, norank_f_Oscillospiraceae, Negativibacillus, norank_f_UCG-010, Anaeroplasma, DTU089, UCG-009, and Lachnospiraceae_UCG-010* (Figure 7B). Additionally, pairwise comparisons by Mann-Whitney U test showed that the relative abundance of *Alistipes* in the LPS group was significantly higher than that in the SS group (*P* < 0.05). Following EP-Se supplementation, the relative abundances of *Christensenellaceae_R-7_group, UCG-005, norank_f_Oscillospiraceae, DTU089*, and *norank_f_UCG-010* were significantly increased compared with the LPS group (*P* < 0.05). Following SS supplementation, the relative abundance of *Negativibacillus* was significantly increased compared with the LPS group (*P* < 0.05) (Figure 7C).

### 3.8 Effects of EP-Se on SCFA concentrations in LPS-challenged broilers

**FIGURE 8:**
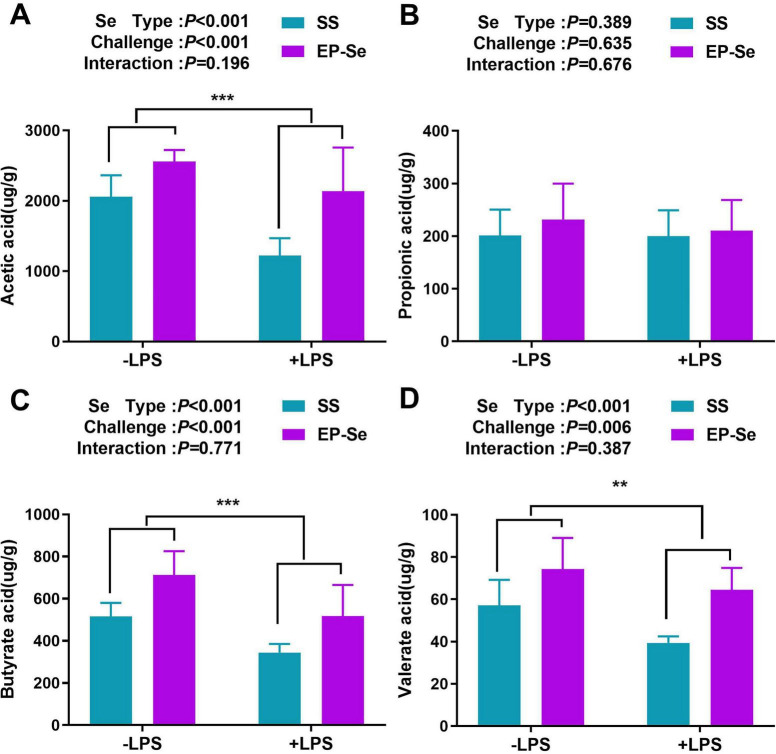
Effects of EP-Se on SCFAs (short chain fatty acids) in cecal contents of LPS-challenged broilers. Results analysis of acetic acid **(A)**, propionic acid **(B)**, butyric acid **(C)**, and valeric acid **(D)**. Values are means (*n* = 6) with their standard errors represented by vertical bars. **P < 0.01, ***P < 0.001 showed that the main effect of LPS was significant.

As shown in Figure 8, the concentrations of acetic acid, butyric acid, and valerate in the cecal microbiota community reduced significantly after LPS stimulation. However, EP-Se significantly increased the concentrations of acetic acid, butyric acid, and valerate in the cecal microbiota community (*P* < 0.05).

## 4 Discussion

In modern intensive broiler farming, the incidence of oxidative stress and immune stress remains high, leading to decreased growth performance, compromised immune function, and deterioration of product quality, which causes severe economic losses (23). Nutritional intervention is an important strategy to alleviate these stresses. Among essential trace elements, selenium plays a central role in maintaining redox homeostasis, immune function, and intestinal health through its participation in the synthesis of 25 selenoproteins (9, 24). Previous studies have demonstrated that oxidative and immune stressors significantly impair performance: overactivation of inflammatory pathways elevates proinflammatory mediators, thereby reducing productivity (25), while oxidative stress during days 16–20 markedly reduces ADG and ADFI and increases FCR (26). Compared with inorganic selenium, organic selenium exhibits higher bioavailability and safety, enabling more effective physiological functions (10, 13, 27, 28). In this study, we evaluated the protective role of EP-Se against LPS-induced oxidative and immune stress in broilers. The results showed that EP-Se supplementation was more effective than sodium selenite in alleviating LPS-induced growth inhibition, oxidative stress, and intestinal injury, highlighting the superior efficacy of organic selenium under stress conditions.

Selenium serves as an essential component of selenoproteins such as GSH-Px and TrxR, playing a pivotal role in scavenging ROS and alleviating lipid peroxidation (29). Rana (30) demonstrated that dietary supplementation with appropriate levels of selenium significantly enhanced the enzymatic activities of GSH-Px and TrxR, markedly reduced serum levels of H2O2 and MDA, effectively interrupted lipid peroxidation processes, and maintained cell membrane integrity. In the present study, EP-Se enhanced antioxidant defense by upregulating the expression of selenoprotein genes (GPx2, GPx3, GPx4, SELT, and SEL-H) in the liver. Under non-LPS conditions, EP-Se also exhibited favorable antioxidant effects compared to SS, as evidenced by significantly increased antioxidant enzyme activities and decreased MDA concentrations.

The immunomodulatory function of selenium is also prominent. Studies have shown that selenium can effectively mitigate immune suppression and toxic damage induced by heat stress, heavy metals, and mycotoxins through the activation of signaling pathways such as Nrf2 and PI3K/Akt (9). Furthermore, selenium participates in inflammatory responses by regulating the NF-κB signaling pathway, a classical regulator of inflammation whose activation induces the expression of pro-inflammatory cytokines such as IL-6 and IL-8 (31). IL-6, as a pleiotropic inflammatory cytokine, can trigger metabolic disorders, growth inhibition, and immune dysregulation when chronically overexpressed; IL-8 exacerbates tissue inflammatory infiltration and barrier disruption by chemoattracting neutrophils (32, 33). Consistent with previous findings that selenium alleviated *Staphylococcus aureus*-induced bovine mastitis (34) and hepatic inflammatory injury (35) by inhibiting NF-κB pathway activation and reducing pro-inflammatory factor expression, our results demonstrated that EP-Se inhibited the expression of the TLRs/NF-κB signaling pathway and downstream inflammatory genes (IL-6, IL-8, and TNF-α), reducing serum inflammatory cytokine levels, thereby effectively alleviating systemic inflammation in broilers under LPS challenge.

Notably, TLRs serve as pattern recognition receptors and represent key upstream molecules for activating the NF-κB inflammatory pathway; their inhibition can block the amplification of inflammatory signaling cascades (36). Moreover, as the largest immune organ in the body, the intestinal inflammatory state is closely associated with hepatic NF-κB activation. This study observed that EP-Se improved duodenal villus height, increased microbial α-diversity, maintained β-diversity, and attenuated LPS-induced dysbiosis, with enrichment of beneficial bacterial families such as Oscillospiraceae and Lachnospiraceae promoting SCFA production. These findings suggest that EP-Se may further block endotoxin translocation and systemic inflammatory circulation by attenuating local intestinal inflammation and improving mucosal barrier function. Collectively, these results elucidate that EP-Se sustains broiler health and alleviates LPS-induced injury through a multi-target mechanism involving “antioxidation-suppression of the TLRs-NF-κB pathway-protection of intestinal barrier.”

Approximately one-fourth of intestinal bacteria harbor genes encoding selenoproteins (37). Selenium can thus substantially influence gut microbial composition (38). Zhai et al. (39) reported that selenium supplementation decreased the abundance of *Dorea* sp. whereas increasing *Turicibacter* and *Akkermansia*, both associated with beneficial metabolic activity. In this study, we found that EP-Se exerted significant regulatory effects on the intestinal microbiota. Regarding diversity, EP-Se significantly increased α-diversity and β-diversity under LPS challenge conditions, and ameliorated LPS-induced dysbiosis; under non-LPS conditions, EP-Se also enhanced α-diversity compared to SS. Regarding community composition, under LPS challenge conditions, EP-Se significantly modulated the relative abundances of *Firmicutes* and *Bacteroidetes* at the phylum level, reduced the abnormal elevation of *Alloprevotella* at the genus level, and concurrently increased the abundances of *Oscillospiraceae* and *Lachnospiraceae*. *Firmicutes* and *Bacteroidetes* are the two most abundant bacterial phyla in the intestine, and their ratio (F/B ratio) is considered an important indicator reflecting intestinal microecological balance (40). LPS treatment typically leads to F/B ratio dysregulation and promotes the expansion of pro-inflammatory bacterial communities, whereas EP-Se intervention helps restore this balance, suggesting that it may maintain intestinal homeostasis by reshaping the phylum-level bacterial community structure. At the genus level, EP-Se significantly enriched multiple genera with important physiological functions. Among these, the increased abundance of *Faecalibacterium*, one of the most abundant butyrate-producing bacteria in the intestine, is particularly noteworthy. This genus provides energy sources for colonic epithelial cells through butyrate production, enhances intestinal barrier function, and exhibits significant anti-inflammatory effects; its reduced abundance is closely associated with various inflammatory bowel diseases and metabolic syndromes (41). Additionally, as a classic probiotic genus, the enhanced abundance of *Lactobacillus* by EP-Se indicates that selenium may enhance host defense capacity by promoting lactic acid bacteria proliferation. Lactic acid bacteria not only produce lactic acid to lower intestinal pH and inhibit pathogens, but also regulate host immune responses through activation of pattern recognition receptors (42). Meanwhile, EP-Se also increased the abundance of *Alistipes*, a strictly anaerobic bacterium belonging to the phylum Bacteroidetes that is a common commensal in the intestine with anti-inflammatory and immunomodulatory properties; its production of succinate and short-chain fatty acids helps maintain the stability of the intestinal microenvironment (43). *Alloprevotella* is known to correlate with inflammatory and metabolic disorders (44), whereas *Lachnospiraceae* (e.g., *Ruminococcus*) can attenuate intestinal inflammation through butyrate production (45). These findings indicate that EP-Se synergistically promotes the optimization of intestinal microbiota structure and thereby improves intestinal health through multi-level regulation of key functional genera (including butyrate-producing bacteria *Faecalibacterium* and *Lachnospiraceae*, probiotic *Lactobacillus*, and anti-inflammatory bacterium *Alistipes*) and core bacterial phyla (*Firmicutes* and *Bacteroidetes*). Importantly, these EP-Se-enriched bacterial genera, particularly *Faecalibacterium* and *Lachnospiraceae*, serve as the primary producers of butyrate in the intestinal tract. This establishes a critical link between EP-Se-induced microbiota modulation and subsequent metabolic outcomes, setting the stage for SCFA-mediated intestinal protection.

SCFAs, particularly butyrate, are pivotal mediators linking gut microbiota to host immune homeostasis and intestinal integrity (46). This study demonstrates that EP-Se significantly enriched butyrate-producing bacteria (*Faecalibacterium* and *Lachnospiraceae*) and elevated intestinal SCFA levels—including acetate, butyrate, and valerate under LPS challenge, and butyrate alone under basal conditions. Mechanistically, increased butyrate inhibits histone deacetylases in immune cells, thereby suppressing NF-κB-mediated production of pro-inflammatory cytokines (IL-6, IL-8, and TNF-α) while enhancing epithelial proliferation and tight junction protein expression (47). Valerate further contributes to immune modulation via Th17 pathway regulation (48). These findings establish a coherent axis: EP-Se drives enrichment of butyrate producers, elevates luminal butyrate, attenuates mucosal inflammation, and ultimately improves duodenal architecture and barrier function. Thus, EP-Se exhibits substantial potential as an intestinal health promoter in broiler production.

## 5 Conclusion

In summary, EP-Se alleviated hepatic and intestinal oxidative stress and inflammation via modulation of redox-sensitive signaling pathways. These protective effects were further linked to improved gut homeostasis, as EP-Se enriched butyrate-producing bacteria (*Faecalibacterium* and *Lachnospiraceae*), elevated intestinal butyrate levels, and consequently reduced proinflammatory cytokine production (IL-6, IL-8, TNF-α) while enhancing duodenal villus architecture. These findings establish EP-Se as a promising functional organic selenium additive for promoting antioxidant capacity and intestinal health in broiler production.

## Data Availability

The raw data supporting the conclusions of this article will be made available by the authors, without undue reservation.
